# Comparative Analysis of Cellular Immune Responses in Treated *Leishmania* Patients and Hamsters against Recombinant Th1 Stimulatory Proteins of *Leishmania donovani*

**DOI:** 10.3389/fmicb.2016.00312

**Published:** 2016-03-22

**Authors:** Sumit Joshi, Narendra K. Yadav, Keerti Rawat, Chandra Dev P. Tripathi, Anil K. Jaiswal, Prashant Khare, Rati Tandon, Rajendra K. Baharia, Sanchita Das, Reema Gupta, Pramod K. Kushawaha, Shyam Sundar, Amogh A. Sahasrabuddhe, Anuradha Dube

**Affiliations:** ^1^Parasitology Division, Council of Scientific and Industrial Research–Central Drug Research InstituteLucknow, India; ^2^Department of Medicine, Institute of Medical Sciences, Banaras Hindu UniversityVaranasi, India; ^3^Molecular and Structural Biology Division, Council of Scientific and Industrial Research–Central Drug Research InstituteLucknow, India

**Keywords:** *Leishmania donovani*, recombinant Th1 stimulatory proteins, immunogenicity, cellular response, lymphocytes, PBMCs, treated *Leishmania*-infected hamster, treated *Leishmania* patients

## Abstract

Our prior studies demonstrated that cellular response of T helper 1 (Th1) type was generated by a soluble antigenic fraction (ranging from 89.9 to 97.1 kDa) of *Leishmania donovani* promastigote, in treated *Leishmania* patients as well as hamsters and showed significant prophylactic potential against experimental visceral leishmaniasis (VL). Eighteen Th1 stimulatory proteins were identified through proteomic analysis of this subfraction, out of which 15 were developed as recombinant proteins. In the present work, we have evaluated these 15 recombinant proteins simultaneously for their comparative cellular responses in treated *Leishmania* patients and hamsters. Six proteins *viz.* elongation factor-2, enolase, aldolase, triose phosphate isomerase, protein disulfide isomerase, and p45 emerged as most immunogenic as they produced a significant lymphoproliferative response, nitric oxide generation and Th1 cytokine response in PBMCs and lymphocytes of treated *Leishmania* patients and hamsters respectively. The results suggested that these proteins may be exploited for developing a successful poly-protein and/or poly-epitope vaccine against VL.

## Introduction

Visceral leishmaniasis (VL), a life-threatening systemic disease also referred as black sickness or kala-azar, is caused by *Leishmania* (L.) *donovani* and *L. infantum* (chagasi) and is transmitted to the human host *via* the bite of infected female dipteran vector, sandfly. This disease is widespread in the Indian subcontinent, East Africa, Mediterranean basin, Central and South America. About 90% of the annual 100 000 cases of VL are from India, Sudan, Brazil, Ethiopia, Bangladesh, and Nepal. Human migration and environmental changes lead to further expansion of the geographical range of this disease which ultimately affects the epidemiological triad (Desjeux, 2004). In India, Bihar serves as the major epicenter of this disease as it covers 80% of VL cases (Hasker et al., 2012). Available chemotherapeutics proved to be inadequate to curb this disease due to its toxicity and are also not cost effective (Coler et al., 2015). Current kala-azar control roadmap in the Indian subcontinent is threatened by the occurrence of relapse cases (Croft et al., 2006; Srivastava et al., 2011; Garcia-Hernandez et al., 2012; Mohapatra, 2014). Immune response, in particular, cell-mediated immune (CMI) response, is severely compromised during active VL, therefore, its up-regulation is indispensable for the parasite’s clearance (Stanley and Engwerda, 2007). A suitable vaccine against VL offers a viable alternative keeping in view the fact that *Leishmania*-infected individuals generate life-long immunity. Also, considering the existence of 80–90% of subclinical or asymptomatic *Leishmania* cases (endemic healthy contacts) as well as post kala-azar dermal leishmanoid (PKDL) cases, a safe and effective vaccine will be critical if the success of recent VL control efforts in Indian subcontinent is to be sustained (Engwerda and Matlashewski, 2015).

For the reduction of parasitic burden in infected individuals, generation of interleukin (IL)-2, interferon (IFN)-γ and tumor necrosis factor (TNF)-α, indicative of T helper 1 (Th1) type response is essential. Also, there requires a balance between proinflammatory IFN-γ/TNF-α and regulatory IL-10 cytokines (Coler et al., 2015). Leishmanial antigens with predominant Th1 type response in infected rodent models have been recognized as ‘potential protective antigens’ and, therefore, promising vaccine candidates. Based on this, several antigens were evaluated which shows mild to moderate protection in different animal models (mice and hamster) and human subjects as reviewed by Joshi et al. (2014). Moreover, current strategies for vaccine development have advanced toward immunologically vital miniature antigenic regions, i.e., the epitopes identified from potential native or recombinant proteins as they are competent enough to generate protective immunity against infectious organisms. Furthermore, designing of vaccines containing multiple epitopes derived from different antigens, i.e., poly-epitope vaccines fortify the immune response *via* targeting multiple antigenic regions. Also, due to the genetic polymorphism of the mammalian immune system, a multi-component vaccine thought to elicit a better protective immune response (Goto et al., 2011).

Our earlier studies, using classical activity based fractionation and sub-fractionation of the soluble proteins from an Indian *L. donovani* promastigote, led to the identification of a potential sub-fraction (89.9–97.1 kDa) which induced Th1 type cellular response in peripheral blood mononuclear cells (PBMCs)/lymphocytes of treated *Leishmania* patients and hamsters. Furthermore, this fraction also offered significant prophylactic efficacy in hamsters against *Leishmania* challenge (Garg et al., 2006; Kumari et al., 2008a,b). Subsequently, 18 Th1 stimulatory proteins were identified through proteomic characterization of this subfraction (Kumari et al., 2008b). Of these, 15 could be developed as recombinant proteins, some of which were subjected to biochemical and immunological characterization and were assessed for their suitability as prophylactic vaccine in hamster model (Kushawaha et al., 2011, 2012a,b; Gupta et al., 2012, 2014; Jaiswal et al., 2014; Khare et al., 2014; Baharia et al., 2015). In the present communication, all the recombinant proteins were evaluated simultaneously for their comparative immunogenicity (cellular responses) in PBMCs and lymphocytes of treated *Leishmania* patients as well as hamsters in order to identify the most potent ones which can be further taken up for developing potential poly-protein and/or poly-epitope vaccine against VL.

## Materials and Methods

### Host and Parasite

Golden hamsters (*Mesocricetus auratus*, 50–55 g) housed in the climatically controlled room, served as a suitable experimental host. The care and use of hamsters were monitored by institutional animal ethics committee (IAEC) which also approved animal experimentation protocols (Approval No. 150/09/Para/IAEC dated 23.10.09) following guidelines of committee for the purpose of control and supervision of experiments on animals (CPCSEA). *L. donovani* strain (MHOM/IN/80/Dd8) was procured as promastigotes from American type culture collection (ATCC, Manassas, VA, USA) and was maintained *in vitro* following the protocol of Garg et al. (2005). Parasite’s virulence was maintained by serial passaging of amastigote in hamsters (Dube et al., 2005).

### Soluble *L. donovani* (SLD) Promastigote Antigen and Recombinant Proteins

The preparation of SLD antigen from metacyclic promastigotes was done as per the method of Gupta et al. (2007) and stored at -80°C till further use. Fifteen recombinant Th1 stimulatory proteins utilized in this study namely elongation factor-2 (elF-2), p45, aldolase (Aldo), enolase (Eno), triosephosphate isomerase (TPI), protein disulfide isomerase (PDI), cofactor-independent phosphoglycerate mutase (iPGM), adenosyl homocysteinase (ADHT), calreticulin (Cal), heat shock proteins (Hsp) – Hsp70 and Hsp83, trypanothione reductase (TPR), NAD-dependent deacetylase SIR2 homolog (NAD) and two hypothetical proteins (Hypo 1 and 2) have been successfully cloned, expressed and purified. Sequences of all the 15 proteins have been submitted to the national center for biotechnology information (NCBI) as depicted in **Table 1**.

**Table 1:** List of recombinant proteins with their accession no. submitted to the national center for biotechnology information (NCBI).

S. no.	Th1 stimulatory proteins	Accession no.	Approximately weight (in kDa)	Nucleotide BLAST	
				*L. major*	*L. infantum*
1	Calreticulin	EU723848	38	95	99
2	Protein disulfide isomerase	EU723849	55	94	99
3	Enolase	EU723850	50	97	99
4	p45	EU723851	45	97	99
5	Triose phosphate isomerase	EU867389	28	97	99
6	Elongation factor-2	EU929069	70	99	99
7	Aldolase	GQ 220750	46	94	97
8	Trypanothione reductase	JN007485	55	79	79
9	Adenosyl homocysteinase	GU353334	52	88	88
10	Cofactor-independent phosphoglycerate mutase	HM044147	67	94	94
11	NAD-dependent deacetylase SIR2 homolog	JN790591	45	95	98
12	Heat shock protein 70	HQ011382	60	97	98
13	Heat shock protein 83	HQ230577	83	90	90
14	Hypothetical protein 1	JF957190	55	88	90
15	Hypothetical protein 2	JX855827	150	92	99

### Isolation of Lymph Node Cells from Treated *Leishmania*-Infected Hamsters

A total of 40 hamsters were utilized in this study. A 0.1 ml of inoculum containing 10^7^ amastigotes/ml was injected intracardially in 30 hamsters while rest of the 10 animals were kept as uninfected control. The magnitude of infection in infected animals were assessed 1 month later by performing a splenic biopsy. Miltefosine (Synphabase, Switzerland), an oral antileishmanial drug, was administered in 15 out of 30 infected hamsters @ 40 mg/kg bodyweight daily for 5 days. Remaining 15 animals were kept as infected control. The method of Garg et al. (2005) was used for isolation of mononuclear lymph node cells.

### Isolation of PBMCs from Treated VL Patients

Human ethics committee of the kala-azar medical research center (KMRC), Muzaffarpur approved the protocols and study on human subjects belonging to hyper-endemic areas of Bihar (Approval No EC-KAMRC/Vaccine/VL/2007-01). A written informed consent was also taken from all of them before their enrollment to this study. All the study subjects, as shown in **Table 2**, were examined for leishmanial as well as other possible infections by a local clinician. Venous blood samples were collected from one to 6-months-old follow up ambisome or amphotericin B treated VL patients (confirmed by physical examination and clinical symptoms) as well as from healthy contacts (neither showed any clinical symptoms nor received any treatment for kala-azar). PBMCs were isolated from the collected venous blood following the method of Garg et al. (2005).

**Table 2:** Clinical data of treated VL patients and healthy contacts.

S. no.	Gender	Age	Study group	Duration of treatment (approximately in months)
1	Female	45	Healthy contact	–
2	Male	50	Healthy contact	–
3	Female	42	Healthy contact	–
4	Male	22	Healthy contact	–
5	Female	35	Healthy contact	–
6	Male	45	Healthy contact	–
7	Female	45	Healthy contact	–
8	Female	28	Healthy contact	–
9	Male	35	Healthy contact	–
10	Female	20	Cured	6
11	Female	15	Cured	6
12	Female	14	Cured	6
13	Male	15	Cured	6
14	Male	50	Cured	6
15	Male	35	Cured	2
16	Female	20	Cured	3
17	Female	12	Cured	1
18	Female	15	Cured	3
19	Female	40	Cured	1.5
20	Female	30	Cured	1
21	Male	14	Cured	2
22	Male	12	Cured	3.5

### Lymphocyte Proliferation and Nitric Oxide Assay

A 0.1 ml of cell suspension (PBMCs from treated patients/healthy contacts and mononuclear cells from treated/uninfected hamsters) in complete RPMI-1640 (Sigma-Aldrich, USA) containing 1×10^6^ cells/ml was seeded in 96-well flat-bottom tissue culture plates (Nunc, Denmark). Each of the 15 recombinant proteins *viz.* rLdPDI, rLdTPI, rLdelF-2, rLdp45, rLdAldo, rLdEno, rLdiPGM, rLdADHT, rLdCal, rLdHsp70, rLdHsp83, rLdTPR, rLdNAD and the two hypothetical proteins- rLdHypo1 and rLdHypo2 as well as SLD (10 μg/ml/well) were used separately in triplicate. Phytohemagglutinin (PHA, 10 μg/ml, Sigma-Aldrich, USA) for Patient’s PBMCs and concanavalin A (ConA, 10 μg/ml, Sigma-Aldrich, USA) for hamster’s mononuclear cells were used as standard mitogens. Wells without stimulants served as blank controls. Cells were maintained in a CO_2_ incubator (37°C with 5% CO_2_) for 3 days in case of mitogens and for 5 days in case of recombinant proteins. 50 μl of XTT (Biological Industries, Israel) was added to 100 μl of culture supernatant around 18 h prior to termination of the experiment. The absorbance of the samples, at 480 nm with 650 nm as a reference wavelength, was measured in a SPECTRAmax PLUS 384 microplate reader (Molecular Devices, USA).

Similarly, nitric oxide (NO) generation was estimated by co-culturing J774A.1 macrophages with 100 μl supernatant of stimulated mononuclear cells for 24 h and then by adding an equal amount of Griess reagent (Sigma-Aldrich, USA; Garg et al., 2005). The absorbance of the reaction was measured at 540 nm (Ding et al., 1988). Lipopolysaccharide (LPS, 10 μg/ml, Sigma-Aldrich, USA) was used as standard NO producer.

### Estimation of Th1/Th2 Cytokines in Culture Supernatant of PBMCs of Treated Patients/Healthy Contacts

Peripheral blood mononuclear cells (1 × 10^6^ cells/ml) from treated human patients as well as healthy contacts were plated in 96-well culture plates (Nunc, Denmark). Recombinant proteins at a concentration of 10 μg/ml were added to triplicate wells and incubated for 5 days. The levels of IFN-γ, IL-12, TNF-α, IL-10, IL-4, and TGF-β (in pg/ml) were estimated in the culture supernatant by ELISA (enzyme-linked immunosorbent assay) kit (BD OptEIA, Pharmingen).

### Statistical Analysis

Two sets of independent experiments (each containing 3–5 animals) were performed. One-way ANOVA was applied in cell-proliferation assay and NO estimation by comparing recombinant proteins group with SLD in treated hamsters as well as in human patients using GraphPad Prism 6.01 (GraphPad Software, San Diego, CA, USA). Results were expressed as mean ± SE. Significance was considered when the *p*-values were <0.05.

## Results

### Recombinant Th1 Stimulatory Proteins Induce Strong Lymphoproliferative Response in Treated *Leishmania* Hamsters and NO Production in J774 Macrophages

**FIGURE 1:**
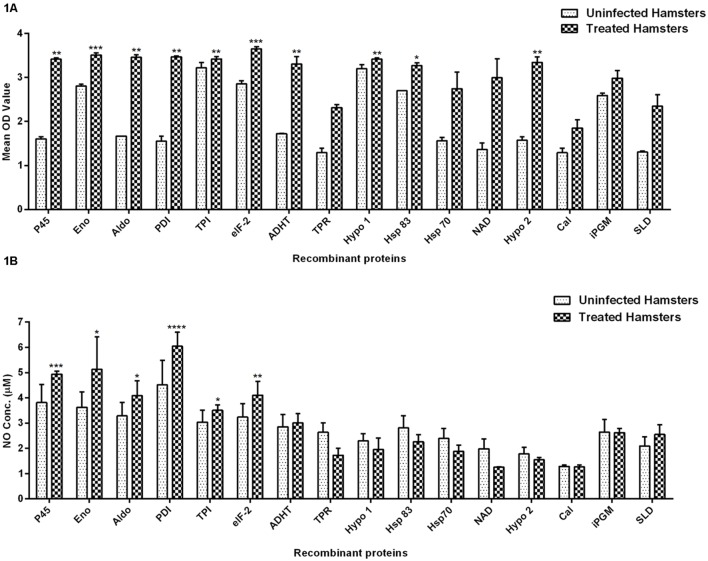
**Lymphoproliferative response and NO production. (A)** LTT response in lymph node cells of treated *Leishmania*-infected hamsters in response to 15 recombinant proteins and SLD (10 μg/ml each) and **(B)** Nitric oxide production measured by Griess reagent in supernatants collected from J774 A.1 macrophage cultures 24 h after incubation. Each bar represents the pooled data (mean ± SE). Significance values indicate the difference in stimulation between the SLD and the recombinant proteins (*p < 0.05, **p < 0.01, ***p < 0.001, and ****p < 0.0001).

The cellular response generated by lymphocytes isolated from lymph nodes of treated hamsters as assessed by XTT against SLD and 15 recombinant proteins have been shown in **Figure 1A**. Significantly, a higher proliferative response against Con A was observed in treated hamsters indicating the procedural sensitivity (data not shown). Ten recombinant proteins *viz*. rLdEno, rLdPDI, rLdTPI, rLdelF-2, rLdp45, rLdAldo, rLdADHT, rHsp83 and the two hypothetical proteins- rLdHypo1 and rLdHypo2 showed significantly higher stimulation in lymphocytes isolated from treated hamsters (mean OD ± SE 3.510 ± 0.04988, 3.468 ± 0.02080, 3.420 ± 0.05009, 3.650 ± 0.04775, 3.415 ± 0.03242, 3.458 ± 0.05535, 3.303 ± 0.1672, 3.272 ± 0.06071, 3.418 ± 0.03132, and 3.343 ± 0.1215) than SLD (mean OD ± SE 2.346 ± 0.2640). Rest of the proteins has shown lesser proliferative response than SLD which was not statistically significant.

Nitric oxide generation by J774 A.1 was studied since it is known that NO-mediated macrophage effector mechanism is critical for reducing parasitic burden in the animal model. NO production was recorded after 24 h of incubation and was found to be significantly higher against six recombinant proteins *viz.* rLdPDI, rLdTPI, rLdelF-2, rLdp45, rLdAldo, and rLdEno in comparison to SLD as shown in **Figure 1B**. NO generation by LPS stimulated cells served as positive control while unstimulated cells are considered as a negative control (data not shown).

### Recombinant Th1 Stimulatory Proteins Stimulate Significant Lymphoproliferative and Predominant Thl Cytokine Response in Treated VL Patients

**FIGURE 2:**
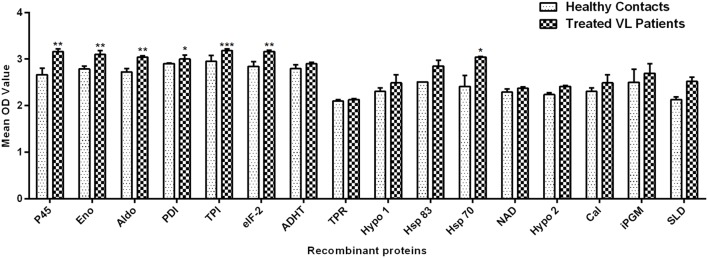
**Lymphoproliferative response in PBMCs of treated VL patients in response to 15 recombinant proteins and SLD (10 μg/ml each).** Each bar represents the pooled data (mean ± SE). Significance values indicate the difference in stimulation between the SLD and the recombinant proteins (*p < 0.05, **p < 0.01, and ***p < 0.001).

Peripheral blood mononuclear cells of treated *Leishmania* patients were further utilized to validate the cellular responses observed in treated hamsters. There were marked differences in responses generated by individual donors in each study group. **Figure 2** depicted the lymphocyte proliferation response generated by 15 recombinant proteins in comparison to SLD. Seven out of 15 proteins, i.e., rLdPDI, rLdTPI, rLdelF-2, rLdp45, rLdAldo, rLdEno, and rLdHsp70 showed significantly higher lymphoproliferative response (3.001 ± 0.08074, 3.179 ± 0.04249, 3.164 ± 0.02774, 3.158 ± 0.06383, 3.038 ± 0.03051, 3.098 ± 0.08782, and 3.044 ± 0.01308) in PBMCs from treated VL patients. The higher proliferative response against PHA indicated procedural sensitivity (data not shown).

Cytokine responses of those recombinant proteins, that have shown optimal lymphoproliferative response and NO generation in both human PBMCs and hamster’s lymphocytes, i.e., rLdEno, rLdAld, rLdp45, rLdTPI, rLdPDI, and rLdelF-2, were measured in PBMCs of treated *Leishmania* patients as well as healthy contacts. The levels of IFN-γ, TNF-α, and IL-12 (Th1 cytokines) were found to be upregulated as compared to Th2 cytokines (IL-4, IL-10, and TGF-β) as shown in **Table 3**.

**Table 3:** Cytokine levels in recombinant proteins and SLD stimulated PBMCs from treated VL patients (T) and healthy contacts (HC).

S. No.	Treatment groups	Th1 cytokines			Th2 cytokines		
		IL-12	IFN-γ	TNF-α	IL-4	IL-10	TGF-β
1	SLD (T)	530.8 (75.88); (320.1–741.5)	127.2 (16.23); (88.82–165.6)	603.6 (63.66); (453.1–754.2)	206.5 (61.25); (61.64–351.3)	287.1 (66.57); (129.7–444.6)	2567 (460.7); (1478–3656)
2	SLD (HC)	170.9 (72.35); (-29.98–371.8)	105.0 (21.77); (44.54–165.4)	157.4 (67.49); (-30.01–344.8)	225.1 (9.752); (198.0–252.2)	44.95 (17.11); (-2.55–92.44)	2191 (291.4); (1382–3000)
3	p45 (T)	833.5 (139.5); (503.5–1163)	281.7 (70.30); (115.5–447.9)	360.3 (104.3); (113.7–606.8)	204.7 (55.81); (72.67–336.6)	322 (32.76); (244.5–399.4)	2758 (387); (1843–3673)
4	p45 (HC)	214.4 (117.9); (-112.9–541.6)	502.0 (162.0); (52.35–951.7)	204.4 (65.67); (22.04–386.7)	234.3 (18.25); (183.7–285.0)	23.33 (6.862); (4.275–42.38)	2314 (376.7); (1268–3360)
5	Eno (T)	668.6 (162.4); (284.7–1053)	226.4 (36.63); (139.8–313.0)	734.2 (206.5); (245.9–1222)	161.8 (37.02); (74.26–249.3)	304.3 (42.8); (203.1–405.5)	2712 (403.8); (1757–3666)
6	Eno (HC)	492.1 (156.3); (58.18–926.1)	435.9 (161.2); (-11.65–883.4)	193.4 (43.43); (72.84–314.0)	197.8 (24.88); (128.7–266.9)	42.49 (9.393); (16.41–68.57)	2773 (263.6); (2042–3505)
7	Aldo (T)	1389 (131.2); (1079–1699)	284.6 (88.65); (74.97–494.2)	769.0 (224.2); (238.9–1299)	208.0 (43.50); (105.1–310.8)	294.1 (33.08); (215.9–372.3)	3278 (170.9); (2873–3682)
8	Aldo (HC)	165.4 (24.46); (97.46–233.3)	328.0 (106.2); (33.24–622.7)	405.5 (99.87); (128.2–682.7)	271.5 (21.41); (212.0–330.9)	39.13 (7.734); (17.66–60.6)	2698 (590); (1060–4336)
9	TPI (T)	621.4 (119.2); (339.6–903.2)	301.7 (60.08); (159.6–443.7)	724.9 (164.9); (334.9–1115)	190.9 (57.15); (55.71–326.0)	253.6 (26.93); (189.9–317.3)	2547 (457.4); (1465–3629)
10	TPI (HC)	210.9 (84.67); (-24.15–446.0)	241.1 (52.63); (95.0–387.3)	289.1 (63.22); (113.5–464.6)	239.8 (11.78); (207.0–272.5)	26.15 (4.724); (13.03–39.26)	2733 (786.2); (550.5–4916)
11	PDI (T)	644.9 (46.05); (536.0–753.8)	386.9 (100.3); (149.7–624.1)	886.5 (249.3); (297–1476)	191.0 (54.32); (62.55–319.5)	265.7 (42.08); (166.2–365.1)	2679 (415.8); (1695–3662)
12	PDI (HC)	185.4 (76.55); (-27.16–397.9)	340.6 (93.41); (81.21–599.9)	200.3 (55.16); (47.10–353.4)	222.0 (17.58); (173.2–270.8)	50.4 (12.18); (16.59–84.21)	1705 (160); (1260–2149)
13	elF-2 (T)	571.0 (116.3); (296.0–846.0)	310.4 (82.51); (115.3–505.5)	837.6 (204.3); (354.5–1321)	228.5 (67.02); (69.97–386.9)	272.7 (34.27); (191.7–353.8)	2515 (490.7); (1355–3675)
14	elF-2 (HC)	204.8 (98.27); (-68.03–477.7)	370.3 (160.4); (-75.09–815.6)	321.4 (90.38); (70.43–572.3)	222.9 (9.96); (195.2–250.5)	32.91 (8.252); (9.998–55.82)	3818 (1125); (695.6–6940)

*Values are displayed as mean (SE); (95% confidence interval).*

## Discussion

Successful recovery from VL due to *L. donovani* is associated with the development of lifelong immunity and resistance to reinfection. In view of this fact, that generation of a strong CMI response play a pivotal role in controlling VL, T-cell stimulatory antigens were thought to be good vaccine targets (Kamhawi et al., 2014). Exploration of such antigens from Indian *L. donovani* parasite resulted in the identification of F2 fraction (ranging from 89.9 to 97.1 kDa) that elicited cellular responses in PBMCs/lymphocytes of treated *Leishmania* patients/hamsters (Kumari et al., 2008a). Further, proteomic analysis of F2 fraction revealed 18 proteins (Kumari et al., 2008b) and out of these, 15 *viz.* elF-2, p45, Aldo, Eno, TPI, PDI, iPGM, ADHT, Cal, Hsp70, Hsp83, TPR, NAD, Hypo 1 and 2 were developed as recombinant ones. Most of these recombinant proteins were individually characterized biochemically and immunologically as potential vaccine candidates (Kushawaha et al., 2011, 2012a,b; Gupta et al., 2012, 2014; Jaiswal et al., 2014; Khare et al., 2014; Baharia et al., 2015). However, to select the most potential one(s) for further advancement toward the development of an effective poly-protein vaccine against VL, all these recombinant Th1 stimulatory proteins were simultaneously evaluated for their immunogenicity in PBMCs/lymphocytes of treated *Leishmania* patients/hamsters. The observation made herein indicated toward the potentiality of seven recombinant proteins *viz*. Eno, Aldo, p45, TPI, PDI, elF-2, and Hsp70 which was well-supported by the findings in other parasitic and infectious diseases highlighting them as a suitable vaccine candidates (Palm et al., 2003; Pal-Bhowmick et al., 2007; Avilan et al., 2011; Li et al., 2011; Chen et al., 2012; Matsubayashi et al., 2013; McNulty et al., 2013; Wang et al., 2013; Zinsser et al., 2013).

As stated earlier that due to the genetic polymorphism of the mammalian immune system, a multicomponent vaccine is considered to evoke a better protective immune response. Therefore, as a result, Q protein, Leish-111f, Leish-110f, KSAC, etc., came into existence that have been demonstrated to afford better protection against experimental VL (Molano et al., 2003; Parody et al., 2004; Coler et al., 2007; Trigo et al., 2010; Goto et al., 2011). Among these polyprotein vaccines, Leish-110f is under clinical trial in Indian population and the outcome of this vaccination trial is yet to be seen. However, since, the strains of *L. donovani* in East Africa and *L. infantum* in the Mediterranean Basin are markedly heterogeneous and genetically different from Indian strains (Abass et al., 2015) it is imperative to design and develop an indigenous polyprotein/polytope vaccine.

Since, *L. donovani* infection in Syrian golden hamster closely mimics systemic infection in human, producing similar clinicopathological symptoms, the animal represents as an ideal model for progressive VL (Garg and Dube, 2006). Therefore, the cellular immune response of these recombinant proteins was studied in lymphocytes of treated *Leishmania*-infected hamsters and was further validated in PBMCs of treated VL patients and healthy contacts.

A range of factors may contribute to the immunogenicity of proteins in VL patients but a major driver for immunogenicity is likely to be the T-cells. Therefore, T-cell function is analyzed by LTT as it can indicate toward T-cell activation. In this study 10 out of 15 proteins *viz.* Eno, Aldo, p45, TPI, PDI, elF-2, Hsp83, ADHT, and Hypo 1 and 2 showed a better proliferative response in lymphocytes of treated hamster. Further, since NO is up-regulated by IFN-γ, in the absence of commercially available cytokine reagents for hamsters, the same was estimated indirectly using NO assay. Herein, six out of above stated 10 proteins with the better lymphoproliferative response, i.e., Eno, Aldo, p45, TPI, PDI, and elF-2 have shown optimally good NO response indicating toward their being highly immunogenic.

However, among all the 15 recombinant proteins, only seven proteins *viz.* Eno, Aldo, p45, TPI, PDI, elF-2, and Hsp70 exhibited good T-cell reactivity in PBMCs of treated VL patients and healthy contacts endorsing the observations made in lymphocytes of treated hamster. These six proteins, when further evaluated for their cytokine response in PBMCs, generated Th1 type immune response which was characterized by high production of IFN-γ, IL-12 and TNF-α against the recombinant proteins as compared to SLD and a low production of IL-4 and IL-10 which was inferior to SLD. The LTT response, as well as the cytokine levels in both the study groups, was comparable, suggesting that the exposed population to *Leishmania* infection, develop a strong protective immunity as was also observed by Tripathi et al. (2006).

## Conclusion

This study shows that PBMCs and lymphocytes from treated VL patients and hamsters were able to recognize six potential Th1 stimulatory recombinant *Leishmania* proteins with the production of Th1 type cytokines that are associated with *Leishmania* killing. These findings lead to the first step of selecting superior vaccine targets. Now these proteins can be further subjected to the identification of potential antigenic peptides (epitope) using various immunoinformatic tools that can be better utilized for designing poly-epitope vaccine in order to fortify host immunity against dreaded kala-azar disease. This new vaccine approach to treat VL expands host protective immune responses, making host produce beneficial T-cells that alleviate disease symptoms more quickly.

## Author Contributions

SJ, NKY, KR, CDPT, AKJ, PK, RT, RKB, SD, RG, and PKK purified recombinant proteins. SJ, NKY, KR, CDPT performed the human and animal experiments. SS provides human samples. SJ and AD analyzed the data and wrote the manuscript. SJ, AS, and AD conceived the study.

## Conflict of Interest Statement

The authors declare that the research was conducted in the absence of any commercial or financial relationships that could be construed as a potential conflict of interest.
